# Correction: The Collaborative Image of The City: Mapping the Inequality of Urban Perception

**DOI:** 10.1371/journal.pone.0119352

**Published:** 2015-03-20

**Authors:** The PLOS ONE Staff

## Notice of Republication

This article was republished on January 30, 2015, to correct errors in Table 3. Please download this article again to view the correct version. The originally published, uncorrected article and the republished, corrected article are provided here for reference.

## Supporting Information

S1 File Originally published, uncorrected article. (PDF)

S2 File Republished corrected article. (PDF)
