# Supplementary material for: The Collaborative Image of The City: Mapping the Inequality of Urban Perception
Source: PLoS One. 2013 Jul 24;8(7):e68400. doi: 10.1371/journal.pone.0068400 (PMC3722224; doi:10.1371/journal.pone.0068400)
Supplement: File S2 — Supplementary material. (DOCX) [file pone.0068400.s002.docx]

­Supplementary Material for:

The Collaborative Image of The City: Mapping the Inequality of Urban Perception­

P. Salesses (1), K. Schechtner (1, 2, 3), C.A. Hidalgo (1,4,5)^†^.

(1) The MIT Media Lab, Massachusetts Institute of Technology, Cambridge, MA. 02139.

(2) Mobility Department, Austrian Institute of Technology, Vienna, Austria.

(3) Institute of Urban Design and Landscape Architecture, Vienna University of Technology, Vienna, Austria.

(4) Engineering Systems Division (ESD), Massachusetts Institute of Technology, Cambridge, MA. 02139.

(5) Instituto de Sistemas Complejos de Valparaiso (ISCV), Paseo 21 de Mayo, Valparaiso, Chile.

† To whom correspondence should be addressed [hidalgo@mit.edu](mailto:hidalgo@mit.edu)

**Table of Contents**

Data and Biases 2

Measure (Q-score) 5

Q-Score Distribution 6

Visual Survey Methods 7

Spatial Autocorrelation 9

Crime Data 10

Spatially Filtered Regression 10

Data and Biases

Data Collection Process

Online imagery for Boston and New York City was scraped from Google by requesting random Street View locations within a predefined boundary (drawn by hand using a custom Google Maps application). For each city, we started with a list of 1,000 randomly generated coordinate pairs and made requests to Google for the nearest Street View panorama within 50 meters. If no panorama was found, that location was skipped. If Street View imagery was returned, we manually curated results, rejecting unsuitable images, such as those only showing a brick wall or those having uncharacteristically poor image quality. Selected images had to show roughly 75% of one side of a street and 25% of the other, allowing elements in both the foreground and background to be clearly visible in the same image. Imagery from Linz and Salzburg were manually collected and were selected based on availability of access, time onsite and weather.

The collected images were included in an online site where users were asked to chose between two randomly selected images by answering one of the three questions: “Which place looks safer?” "Which place looks more social-class?" or "Which place looks more unique?" After an image was clicked, the choice of the user was recorded and logged as one vote in a database. Users also had the option to rate images as being perceptually equal by pressing a button placed between the two images. Users were not informed about the location of the images during the process.

Participant Demographics

One important question that needs to be addressed here is the possible measurement biases that might come from the demographic of online participants. To test for this, participants were asked to self-report age and gender after contributing five clicks. Self-reporting was high, with 97.1% of the participants providing answers for age and gender. From these, 76.0% identified themselves as male and 21.1% as female. The median self-reported age was 28 years. Finally, participants were geolocated using their IP addresses and the 7,872 unique IP addresses were located in 91 countries (Table 1s).

We test for the significance of possible biases by comparing the Q-scores estimated using different subsets of participants. We do this for participant’s age (above and below the median), gender (male and female) and location (United States vs Non-United States). As controls, we show the correlations obtained for random subsets of participants of the same size (Figures 1s, 2s, 3s). For example, we compare the correlation of the scores obtained for males and females, with the correlation between the scores obtained for two random samples of participants: one matching exactly the number of female participants in the dataset, and the other one matching exactly the number of male participants. The same procedure was used to create controls for the correlations observed for groups of different ages and for US and non-US locations, as proxied by participants IP-addresses. Overall, we find that the correlations obtained for groups of different demographics are not significantly lower than those obtained for the random controls. This indicates that the results of our sample are not driven by the biases in age, gender or location of the study’s participants.

| Country | Number of Participants |
| --- | --- |
| United States of America | 8574 |
| Spain | 1798 |
| United Kingdom | 1064 |
| Canada | 905 |
| Switzerland | 492 |
| Poland | 419 |
| Germany | 388 |
| Australia | 320 |
| France | 293 |
| Netherlands | 235 |
| Mexico | 199 |
| Argentina | 183 |
| Sweden | 147 |
| Brazil | 144 |
| Austria | 129 |
| Italy | 120 |
| Chile | 106 |
| Denmark | 103 |
| Portugal | 99 |
| Belgium | 95 |

Table 1s. Top 20 countries ordered by number of participants

By Voter Gender


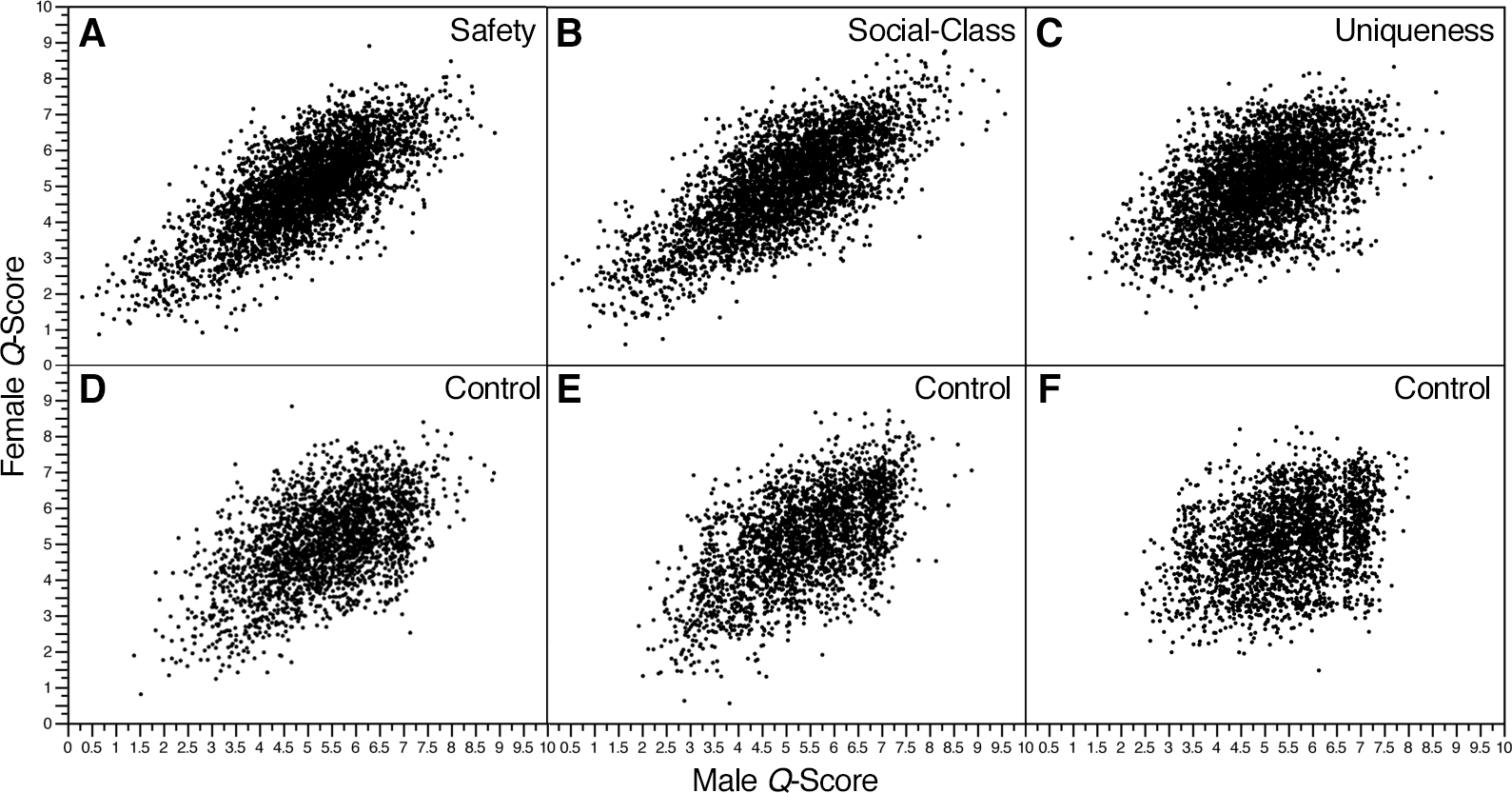


Figure 1s. Scatter plot of *Q*-scores comparing male vs. female votes for A. safety (R^2^=52.0% and p<.0001), B. social-class (R^2^=53.2% and p<.0001), C. uniqueness (R^2^=25.0% and p<.0001), and scatter plots of equal sample sizes (control) for D. safety control (R^2^=28.0% and p<.0001), E. social-class control (R^2^=34.5% and p<.0001), and F. uniqueness control (R^2^=13.3% and p<.0001)

By Voter Age


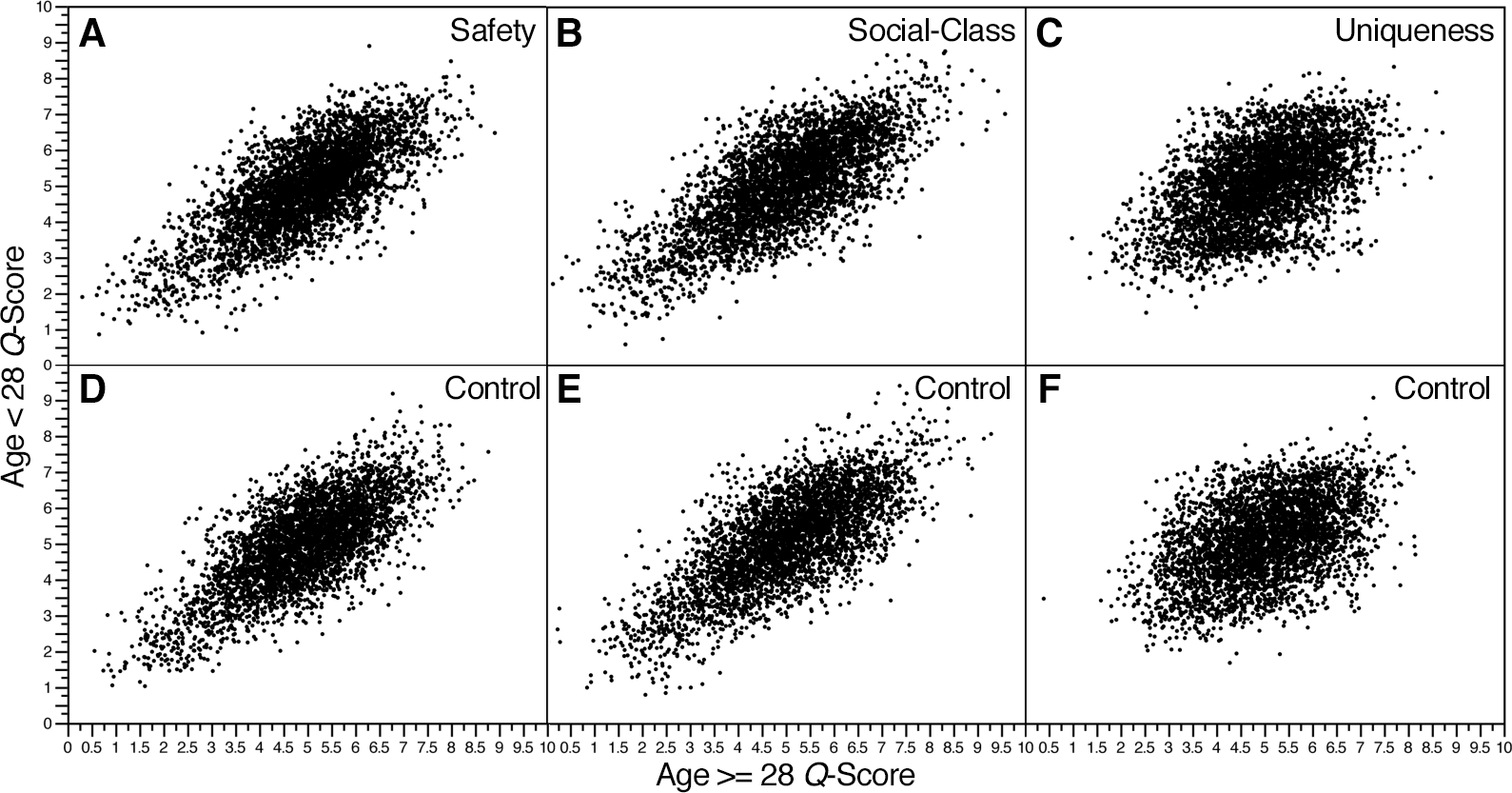


Figure 2s. Scatter plot of *Q*-scores comparing ages above and below the median (28) for A. safety (R^2^=53.7% and p<.0001), B. Social-class (R^2^=54.8% and p<.0001), C. uniqueness (R^2^=23.2% and p<.0001), and scatter plots of equal sample sizes (control) for D. safety control (R^2^=44.8% and p<.0001), E. social-class control (R^2^=40.8% and p<.0001), and F. uniqueness control (R^2^=18.4% and p<.0001)

By Voter Location


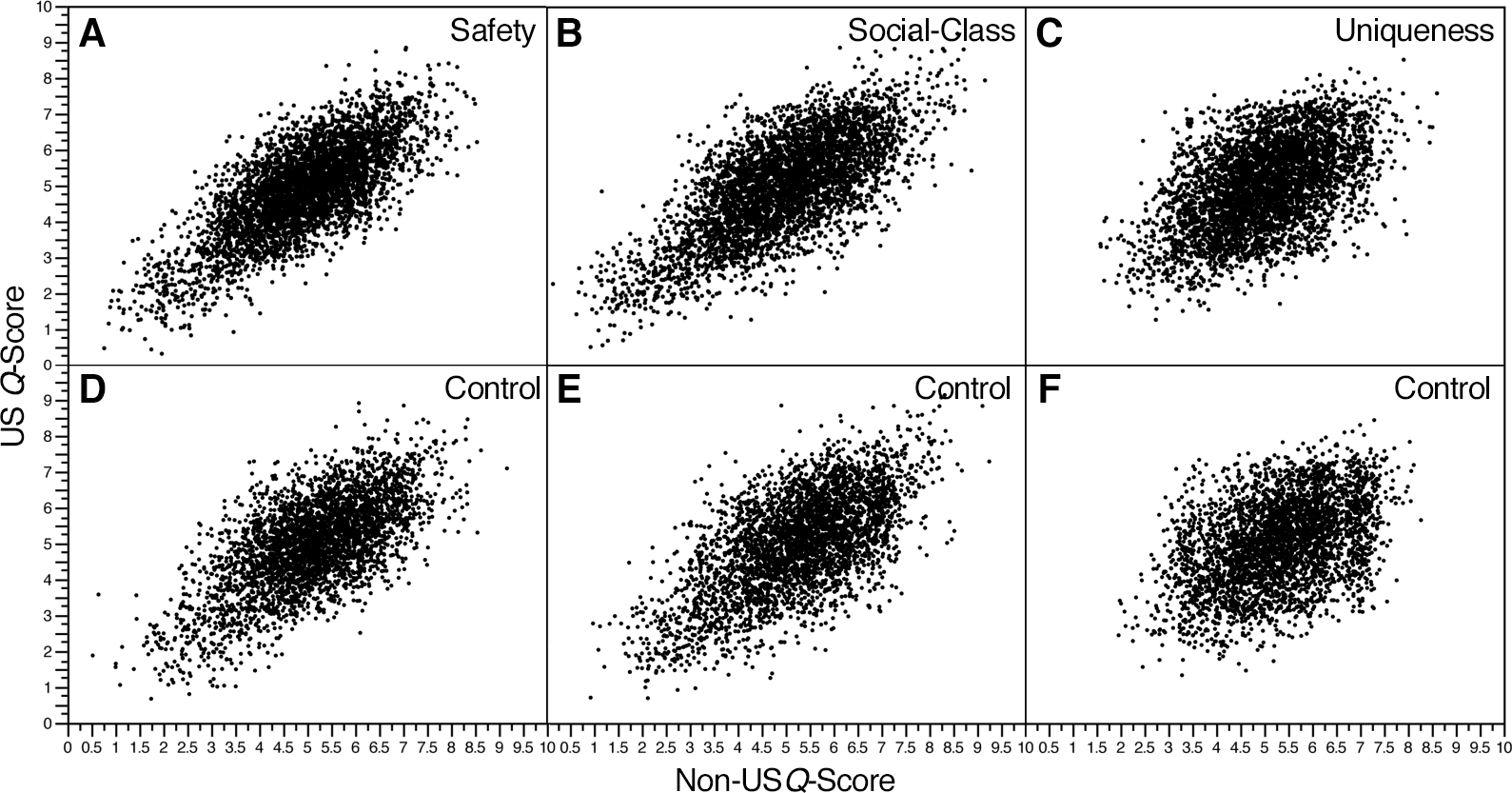


Figure 3s. Scatter plot of *Q*-scores comparing locations inside and outside the United States for A. safety (R^2^=54.4% and p<.0001), B. Social-class (R^2^=50.7% and p<.0001), C. uniqueness (R^2^=24.3% and p<.0001), and scatter plots with equal sample sizes (control) D. safety control (R^2^=44.8% and p<.0001), E. social-class control (R^2^=40.8% and p<.0001), and F. uniqueness control (R^2^=18.4% and p<.0001)

Measure (Q-score)

To compare images across cities, we generated a relative scale that we call Q. Q-scores are calculated based on the voting dataset provided as input, and therefore, cannot be compared with Q-scores obtained for different datasets. For example, if we were to calculate the Q-score for each location in New York City, using only images for NYC, our algorithm would produce a Q-score between 0 and 10 for each image, depending on how that image compared to others. If votes for another city, e.g. Linz, were generated later, there would be no way to compare the score obtained by NYC images with those of Linz, since there would have been no direct, or indirect, comparisons between them.

To address this, we use data on all cities to calculate *Q*-scores. Additionally, we normalize all Q-scores using the theoretical minimum and maximum. We do this by starting with the initial equation for Q:

|  |  | (1) |
| --- | --- | --- |

and plugging in the theoretical best and worst values an image can have. The maximum is obtained for an image that is selected over every image it is paired with, and that was paired with images that were always selected over their pairs:

|  | 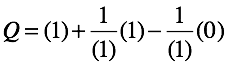. | (2) |
| --- | --- | --- |

This results in a Q-score of 2.

The minimum possible score is obtained for an image that was never selected over its pairs, and that was paired with images that were never selected over their pairs. In this case,

|  | 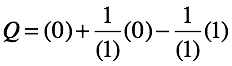 | (3) |
| --- | --- | --- |

which results in a Q-score of -1.

To normalize all equations by the minimum and maximum values, we plug the values of -1 (min q) and 2 (max q) in to the following normalization equation:

|  |  | (4) |
| --- | --- | --- |
|  |  | (5) |

which after simplification,

|  |  | (6) |
| --- | --- | --- |

results in the equation referenced in the main text:

|  |  | (7) |
| --- | --- | --- |

We chose 10 as the Q-score multiplier as a way for the reader to more intuitively understand its values.

Q-Score Distribution

The three figures below show the distribution of Q-scores for each question together with a Gaussian fit (red line). While technically speaking, according to a Liiliefors test only the distribution of uniqueness is strictly Gaussian, we find the distribution of safety and class to be close enough to a Gaussian for the standard deviation to be a good metric of dispersion.


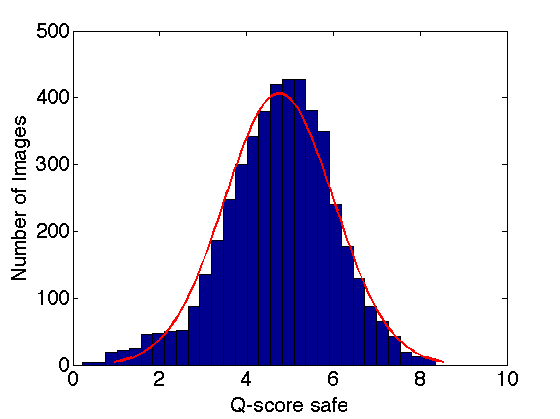

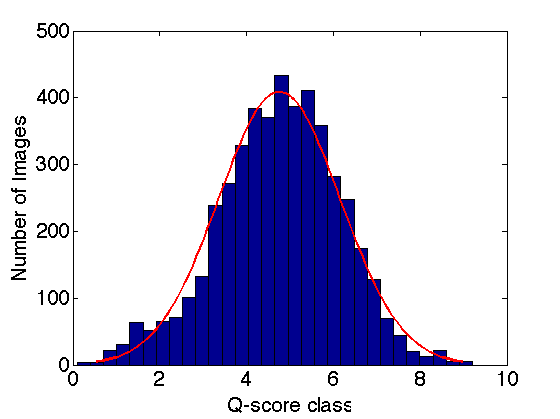

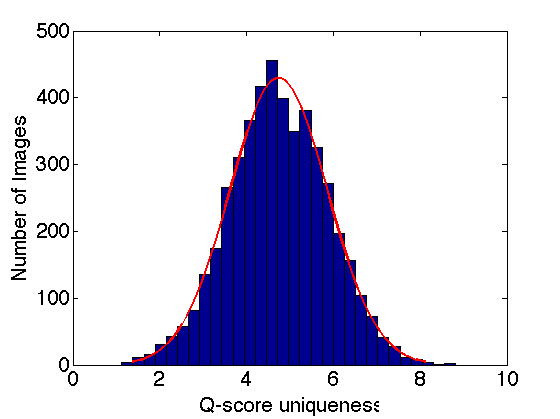


**Figure 4s.** Q-score distribution

Visual Survey Methods

Visual surveys are a group of methods used to measure preferences about a landscape by showing participants images and asking them to score them according to some dimension. For example, in a traditional visual survey, a surveyor would show several images to a participant and ask them to rank the images on some scale, frequently 1-10. Although visual surveys have been used heavily since the 1970’s, surveys are characterized by low numbers of participants and low throughput. The table below summarizes the number of locations, subjects and responses for a large number of visual surveys.

| Authors | Year | Publication | Locations | Subjects | Responses |
| --- | --- | --- | --- | --- | --- |
| Wilson | 1962 | Livability of the City: Attitudes & Urban Development | 34 |  |  |
| Peterson | 1967 | Journal of Regional Science | 100 | 140 | 3,220 |
| Kaplan et al | 1972 | Perception & Psychophysics |  | 88 | 4,928 |
| Herzog et al | 1976 | Environment and Behavior | 86 | 121 | 10,406 |
| Nasar | 1984 | Journal of Cross-Cultural Psychology | 24 | 46 | 1,104 |
| Devlin & Nasar | 1989 | Journal of Environmental Psychology | 40 | 40 | 1,600 |
| Steinitz | 1990 | Landscape & Urban Planning | 48 | 200 |  |
| Hammitt & Patterson | 1994 | Landscape & Urban Planning | 96 | 721 |  |
| Bishop | 1997 | Landscape & Urban Planning | 100 | 59 | 708 |
| Hunker & Kienast | 1999 | Landscape Ecology | 1 | 181 | 1,267 |
| Wherrett | 1999 | Landscape & Urban Planning | 90 | 165 | 3,300 |
| Ayala | 2000 | Landscape & Urban Planning | 176 | 150 |  |
| Cohen et al | 2000 | American Journal of Public Health | 55 |  |  |
| Wherrett | 2000 | Landscape & Urban Planning | 90 | 180 | 3,600 |
| Brown | 2001 | Landscape & Urban Planning | 32 | 60 |  |
| Daniel & Meitner | 2001 | Journal of Environmental Psychology |  | 216 | 10,368 |
| Ross & Mirowsky | 2001 | Journal of Health & Social Behavior |  | 2,482 | 2,482 |
| Arriaza et al | 2003 | Landscape & Urban Planning | 160 | 226 |  |
| De Groot | 2003 | Landscape & Urban Planning |  | 172 |  |
| Dramstad et al | 2006 | Landscape & Urban Planning | 30 | 91 |  |
| Roth | 2006 | Landscape & Urban Planning |  | 321 | 1477 |
| Vandenberg | 2006 | Landscape & Urban Planning | 225 | 500 |  |
| Rogge et al | 2007 | Landscape & Urban Planning | 330 | 130 |  |
| Barga & Bond | 2008 | American Journal of Criminology | 34 |  |  |

Table 2s. A table of visual surveys including the number of locations, subjects and responses.

Spatial Autocorrelation

Spatial autocorrelation measures the similarity of values as a function of their distance. In other words, datasets are spatially autocorrelated if values that are spatially close to each other, have similar qualities and/or attributes.

A popular measure of spatial autocorrelation is Moran’s *I* index, which is defined as

|  | 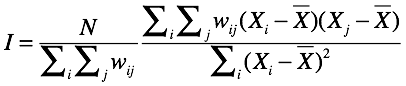 | (1) |
| --- | --- | --- |

where N is the number of spatial elements indexed by i and j; X is the studied attribute;
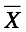
is the mean of *X*; and
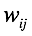
is an element of a spatial weights matrix.

Since Moran’s *I* is an inferential statistic, values are always interpreted within the context of its null hypothesis. For Moran’s *I*, the null hypothesis is complete spatial randomness. Put another way, Moran’s *I* values measure how likely it is for an underlying spatial pattern to be the result of random chance. Moran’s *I* values range from -1 to 1, with negative and positive spatial autocorrelation meaning, respectively, a checkerboard or a totally segregated pattern. Moran’s *I* values near zero indicate that the pattern observed is random.

To understand spatial autocorrelation more intuitively, picture a lattice, where each square has both a location (x,y) and a value (white or black). If the color of a square were the attribute of interest, a standard checkerboard would produce a Moran’s *I* value of -1, indicating perfect anti-correlation of colors (neighbors tend to be opposite). If for instance, all the white squares were on one side of the board, and all the black squares were on the opposite side, the Moran’s *I* value in this case would be close to +1, indicating perfect correlation or spatial segregation (neighbors tend to be equal).

To test a hypothesis statistically, Moran’s *I* values can be transformed into z-scores. When a z-score > 1.96 or < -1.96, spatial autocorrelation is significant at the 5% level. Below are the z-scores and p-values for our dataset, for each city and question in our study.

| City | Question | z-score | p-value |
| --- | --- | --- | --- |
| New York City | Safety | 36.592601 | < 0.000001 |
| New York City | Social-Class | 45.701121 | < 0.000001 |
| New York City | Uniqueness | 48.036719 | < 0.000001 |
| Boston | Safety | 28.911159 | < 0.000001 |
| Boston | Social-Class | 26.848713 | < 0.000001 |
| Boston | Uniqueness | 17.886751 | < 0.000001 |
| Salzburg | Safety | 12.565282 | < 0.000001 |
| Salzburg | Social-Class | 14.318874 | < 0.000001 |
| Salzburg | Uniqueness | 9.621923 | < 0.000001 |
| Linz | Safety | 3.156045 | 0.001599 |
| Linz | Social-Class | 3.618932 | 0.000296 |
| Linz | Uniqueness | 11.726760 | < 0.000001 |

Table 3s. Table of Moran’s *I* values for the four cities and three questions in our study.

Crime Data

We use crime data from the NYPD made available at http://projects.nytimes.com/crime/homicides/map/ in July 11, 2012. The data contains geotagged information on the number of homicides between 2003 and 2011 in the five boroughs of NYC.

Spatially Filtered Regression

We use a Getis Spatially Filtered Regression (GSFR), rather than an Ordinary Least Square (OLS) regression, because the use of OLS is only justified when the residuals of the OLS regression are not spatially autocorrelated (and they are in this case). If the residuals of the regression are spatially correlated, the statistical model is not fully specified, and hence, the model requires the incorporation of additional explanatory variables. In our case, we find that the residuals of the spatially filtered regression do not exhibit spatial autocorrelation (Moran’s *I* *z*-score=-0.23 *p*-value=0.82), implying that the model is fully specified.

Getis Spatially Filtered Regression (GSFR) splits each explanatory variable (x) into two, a spatial (L_x_) and a non-spatial component (x*). This helps produces estimates of the effect of each variable that are separate from its spatial variation.

For each location i, the two variables associated with x are defined as

|  | ${x_{i}}^{*}=\frac{x_{i}W_{i}}{G_{i}(n-1)}$ | (1) |
| --- | --- | --- |
|  | $L_{x_{i}}=x_{i}-{x_{i}}^{*}$ | (2) |

where W_i_=Σ_j_w_ij_ is the sum of the spatial weights (in our case 1/distance between locations i and j), n is the number of locations considered and

|  | $G_{i}=\frac{\sum_{j} w_{ij}x_{j}}{\sum_{j} x_{j}}$ | for j≠i | (3) |
| --- | --- | --- | --- |

In a spatially filtered regression, each explanatory variable is replaced by a non-spatial, starred (*) variable, and a spatial, L component.

Table 4s shows the results for the OLS. The GSFR results are presented in the main text (table 3). Both tables use three groups of variables. First we have the population and area of each zip code, which are expected to be obvious correlate of crime. This is because crime should increase with population (more potential victims and perpetrators) and area (more possible locations where crime could have happened). We note that, since we are using logarithms, the combination of these two also control for population density. The second group of variables is income and age, also in logarithms. Income allows us to control for the wealth of a neighborhood, since crime tends to be more common in poorer areas. Also, younger neighborhoods tend to exhibit more crime, since it is well known that criminal behavior is more prevalent in males between 15 and 30 years of age. Finally, we have variables on urban perception. We note that even in the OLS, the standard deviation in the perception of safety, and class, are significant correlates of the number of homicides, after controlling for income and age.

| OLS | DEPENDENT VARIABLE | |  | Log(Number of Homicides + 1) | | |  |  |
| --- | --- | --- | --- | --- | --- | --- | --- | --- |
|  |  |  |  |  |  |  |  |  |
|  | Log(Population) | Log(Area) | Log(Income) | Log(Age) | Qsafety | SQsafety | Qclass | SQclass |
| Coefficients | 0.7961 | -0.1959 |  |  | -0.0832 | -0.2334 | -0.1497 | 0.2047 |
| t-statistic | 5.9193 | -1.4377 |  |  | -0.8089 | -2.2104 | -1.57 | 1.9266 |
| P-values | 0 | 0.1536 |  |  | 0.4205 | 0.0293 | 0.1195 | 0.0568 |
|  |  |  |  |  |  |  | R^2^ | 0.4799 |
| Coefficients | 0.13798 | 0.5055 | -1.0766 | -1.9467 |  |  |  |  |
| t-statistic | 1.4711 | 4.7807 | -6.3537 | -3.2483 |  |  |  |  |
| P-values | 0.1443 | 0 | 0 | 0.0016 |  |  |  |  |
|  |  |  |  |  |  |  | R^2^ | 0.6663 |
| Coefficients | 0.1093 | 0.5508 | -1.0986 | -2.2215 | -0.1487 | -0.2294 | 0.1409 | 0.1927 |
| t-statistic | 0.9737 | 5.1268 | -5.57 | -3.5709 | -1.8712 | -2.7551 | 1.7422 | 2.3535 |
| P-values | 0.3326 | 0 | 0 | 0.0005 | 0.0642 | 0.007 | 0.0845 | 0.0206 |
|  |  |  |  |  |  |  | R^2^ | 0.6988 |
| All logs in base 10. Area in square kilometers. | | |  |  |  |  |  |  |

Table 4s. Ordinary Least Squares Regression.
