# Supplementary material for: Correction: The Collaborative Image of The City: Mapping the Inequality of Urban Perception
Source: PLoS One. 2015 Mar 20;10(3):e0119352. doi: 10.1371/journal.pone.0119352 (PMC4368184; doi:10.1371/journal.pone.0119352)
Supplement: S1 File — (PDF) [file pone.0119352.s001.pdf]

PLOS ONE

The Collaborative Image of The City: Mapping the Inequality of Urban Perception

Philip Salesses[1], Katja Schechtner[1,2,3], César A. Hidalgo[1,4,5]*

1 The MIT Media Lab, Massachusetts Institute of Technology, Cambridge, Massachusetts, United States of America, 2 Mobility Department, Austrian Institute of Technology, Vienna, Austria, 3 Institute of Urban Design and Landscape Architecture, Vienna University of Technology, Vienna, Austria, 4 Engineering Systems Division, Massachusetts Institute of Technology, Cambridge, Massachusetts, United States of America, 5 Instituto de Sistemas Complejos de Valparaiso, Valparaiso, Chile

Abstract

A traveler visiting Rio, Manila or Caracas does not need a report to learn that these cities are unequal; she can see it directly from the taxicab window. This is because in most cities inequality is conspicuous, but also, because cities express different forms of inequality that are evident to casual observers. Cities are highly heterogeneous and often unequal with respect to the income of their residents, but also with respect to the cleanliness of their neighborhoods, the beauty of their architecture, and the liveliness of their streets, among many other evaluative dimensions. Until now, however, our ability to understand the effect of a city's built environment on social and economic outcomes has been limited by the lack of quantitative data on urban perception. Here, we build on the intuition that inequality is partly conspicuous to create quantitative measure of a city's contrasts. Using thousands of geo-tagged images, we measure the perception of safety, class and uniqueness; in the cities of Boston and New York in the United States, and Linz and Salzburg in Austria, finding that the range of perceptions elicited by the images of New York and Boston is larger than the range of perceptions elicited by images from Linz and Salzburg. We interpret this as evidence that the cityscapes of Boston and New York are more contrasting, or unequal, than those of Linz and Salzburg. Finally, we validate our measures by exploring the connection between them and homicides, finding a significant correlation between the perceptions of safety and class and the number of homicides in a NYC zip code, after controlling for the effects of income, population, area and age. Our results show that online images can be used to create reproducible quantitative measures of urban perception and characterize the inequality of different cities.

Citation: Salesses P, Schechtner K, Hidalgo CA (2013) The Collaborative Image of The City: Mapping the Inequality of Urban Perception. PLoS ONE 8(7): e68400. doi:10.1371/journal.pone.0068400

Editor: Alain Barrat, Centre de Physique Théorique, France

Received November 5, 2012; **Accepted** May 22, 2013; **Published** July 24, 2013

Copyright: © 2013 Salesses et al. This is an open-access article distributed under the terms of the Creative Commons Attribution License, which permits unrestricted use, distribution, and reproduction in any medium, provided the original author and source are credited.

Funding: Massachusetts Institute of Technology Media Lab Consortia Funding. The funders had no role in study design, data collection and analysis, decision to publish, or preparation of the manuscript.

Competing Interests: César A Hidalgo is a PLOS ONE Editorial Board member. This does not alter the authors' adherence to all the PLOS ONE policies on sharing data and materials.

* E-mail: hidalgo@mit.edu

Introduction

In "The Image of The City", Kevin Lynch defines the city as a form of temporal art [1]. Much like sculptures, cities are spatial structures, but unlike sculptures, cities are too large to be experienced in a single try. Hence, people experience cities through unique temporal sequences that are reversed, interrupted and cut-across from the sequences experienced by others. Ultimately, in a world in which people's experiences of urban environments is unique, this uniqueness can give rise to an alternative form of inequality, where differences in the experiences elicited by different neighborhoods, rather than income, becomes an important source of interpersonal contrast.

Neighborhoods often differ in their demographics, such as the income and ethnicity of the people that inhabits them, but also on how safe they feel, how clean they are, how historical they look, and how lively they are, among many other evaluative dimensions [2]. Certainly, many of these dimensions will correlate with measures of income, but income will not necessarily be a complete proxy for all of them. Because of this, it is important to create measures of cities–and their neighborhoods–that incorporate the evaluative aspects of cities that income based measures are unable to fully capture.

In this paper, we present a high-throughput method to quantify people's perception of cities, and their neighborhoods, and use it to measure the perceptual inequality of Boston, New York, Linz and Salzburg. The method is based on image ratings created from the pairwise comparison of images in response to evaluative questions, such as "Which place looks safer?" or "Which place looks more upper-class?" The data shows that the range of perceptions elicited by images from Boston and NYC is wider than the range of perception elicited by the images of Linz and Salzburg. Finally, we validate our measures of urban perception by studying the correlation between urban perception and homicides in New York City, finding a significant correlation between violent crime and urban perception after controlling for income, population, area and age.

We conclude that the method presented in the paper is able to capture information about a city's built environment that is relevant for the experiences of citizens, and not fully contained in income-based measures. Moreover, we conclude that these measures can be used to estimate the contrasts – or inequality – of a city's built environment with respect to these evaluative dimensions.

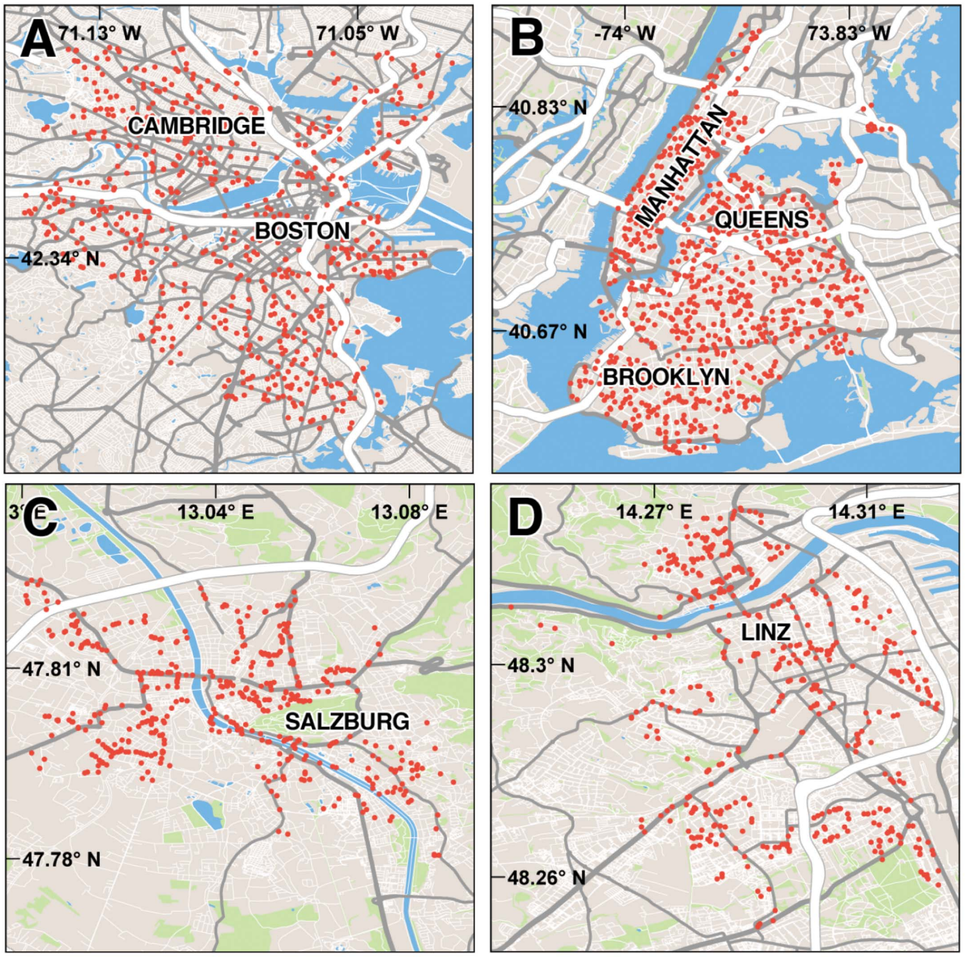

Figure 1. Images used in the study. A–D. Locations from which images were collected for: **A** Boston, **B** New York City, **C** Salzburg and **D** Linz. We note that for many locations, more than one image was collected (with the camera looking in different directions).
doi:10.1371/journal.pone.0068400.g001

A tale of two literatures

Cities, and their neighborhoods, are complex entities that weave together the physical components of the built environment, and the social interactions of the citizens that inhabit them. Yet, the study of cities does not belong to a unified stream of literature, but largely to two parallel branches. On the one hand, we have the literature advanced by urban planners and architects, and on the other, we have the literature advanced by social scientists and natural scientists.

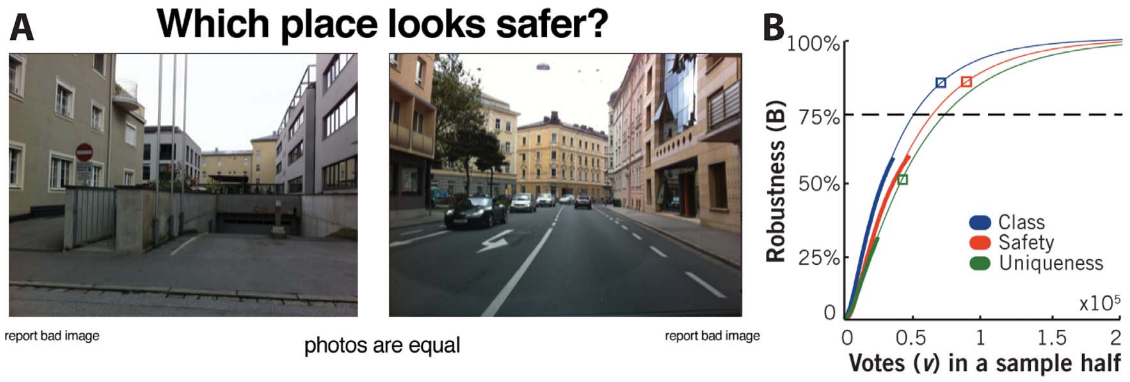

Figure 2. Data Collection Methods. A. The website used to collect votes. Participants were presented a random pair of images and voted by clicking on one in response to the question. **B**. Robustness of the urban perception metric (Q). *B* is the square of the Pearson correlation between two disjoint subsets of votes of size *v* containing the same number of images.
doi:10.1371/journal.pone.0068400.g002

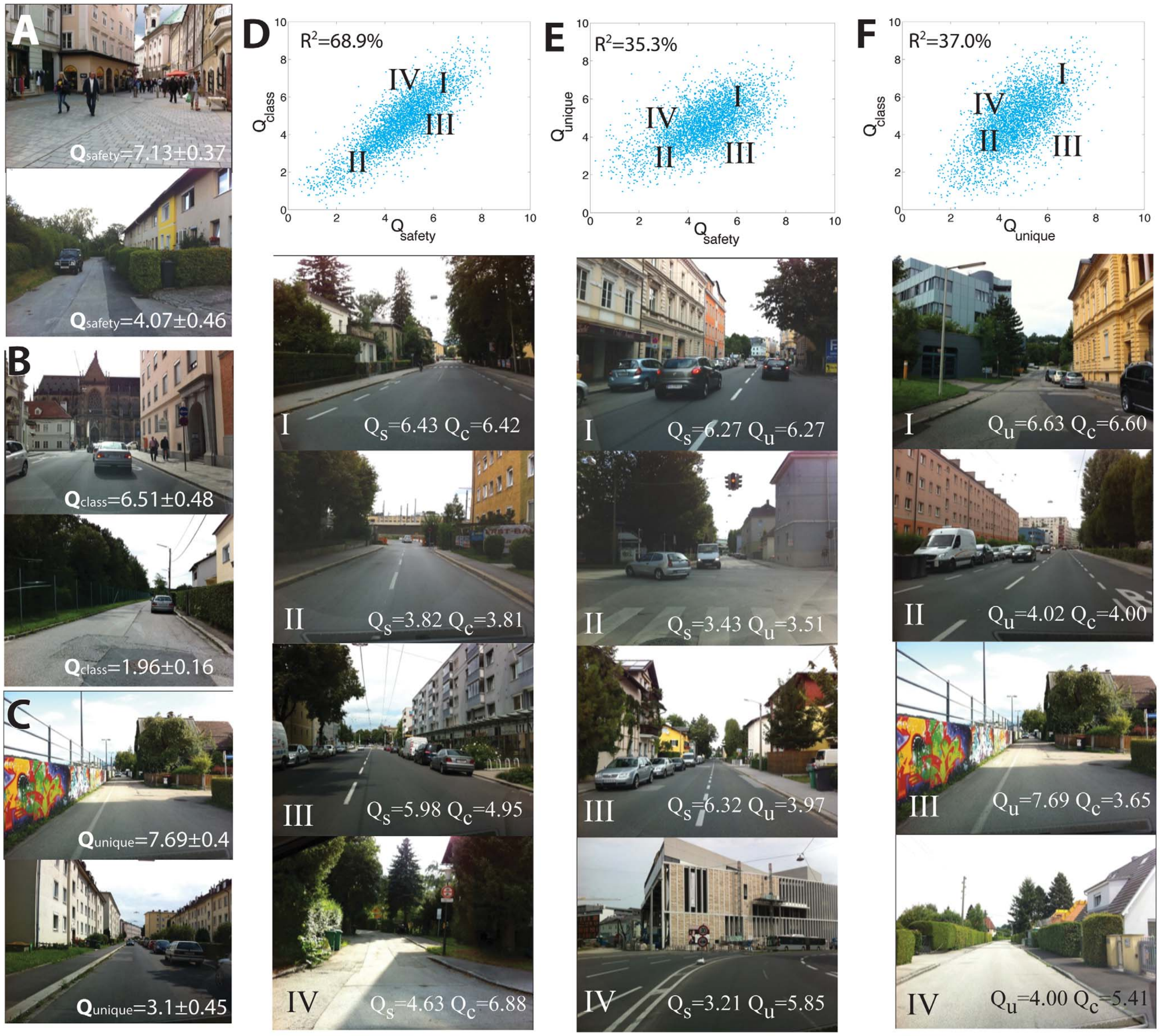

Figure 3. Identifying places associated with different urban perceptions. A. High and low scoring images for safety **B**. social-class and **C**. uniqueness. **D**. Scatter plot of Q-scores for safety and social-class with four examples illustrating images with different combinations of evaluative criteria. **E**. Same as **D**, but for safety and uniqueness. **G**. Same as **D**, but for social-class and uniqueness.
doi:10.1371/journal.pone.0068400.g003

The literature advanced by architects and urban planners puts special emphasis on a city's built environment. During the 20th century, the development of this literature was punctuated by a series of movements, which have resulted in cities combining different architectural and planning styles [3]. Among the most notable of these movements are: the *City Beautiful* or *Civic Art* movement of Charles Mulford Robinson [4], which emphasizes the aesthetic aspects of a city's built environment – think of New York's Grand Central Station; *The Garden City* of Ebenezer Howard [5], which proposed a mixture of low density housing and parks – much like many modern suburbs; and the *Radiant City* of Le Corbusier [3,6], which reconciled Howard's Garden City with high density buildings – NYC Stuyvesant village being an excellent illustration of it.

The literature of architects and urban planners has also been active in the creation of measurements of urban perception along a number of different evaluative dimensions [2]. This study is certainly inspired by these measures, which have been based mostly on visual surveys where people rate images on a 1–10 scale [2,6–14]. The justification of visual surveys is that urban environments have features, such as the exterior beauty of the architecture, or the neatness of the shrubbery, that are not traded in the market. Hence, these cannot be inferred from market mechanisms, such as the price system [2,14–15]. The offline and online studies conducted in the past, however, have lacked the throughput required to make comprehensive maps of urban perception (Table 2s in File S2), and hence, are limited in their ability to compare a large number of cities and neighborhoods.

Within the social sciences, the study of cities has focused mostly on the connection between demographic and economic variables, with the physical appearance of the built environment playing little or no role. The literature advanced by economists, for instance,

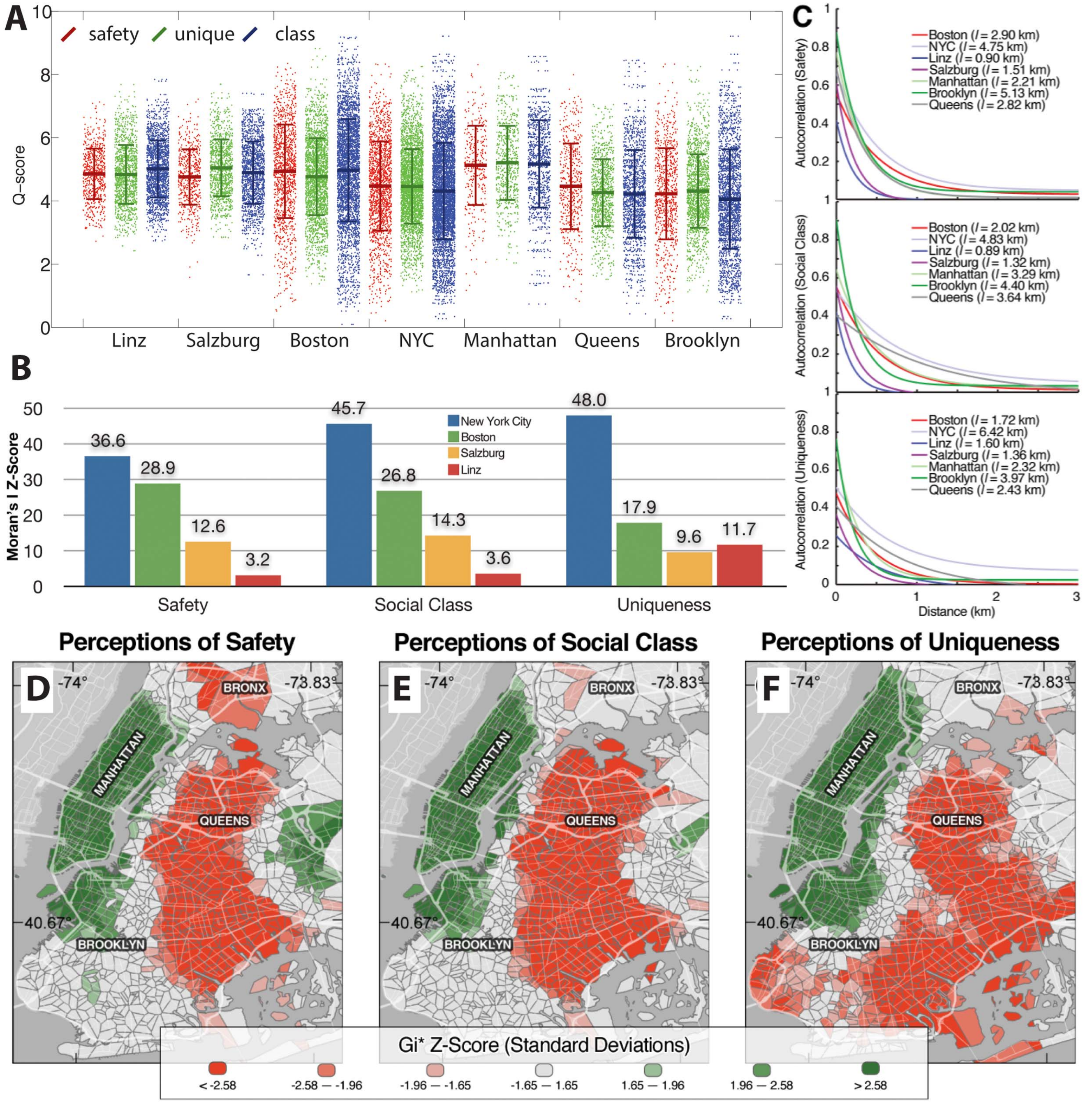

Figure 4. Contrasts in urban perception. A. Scatter plot showing the Q-scores obtained for each image, city and question. Top and bottom whiskers represent one standard deviation. **B**. Moran's I z-scores for each city and question (all p-values$<$0.01, see SM). **C**. Spatial correlograms showing the decay of spatial autocorrelation as a function of distance. **D–F**. Map of NYC showing statistically significant clusters of high -and low- Q-scores for the perception of safety, class and uniqueness according to Getis Gi* statistic. Green shows clusters of positive perceptions (high Q-scores) and red shows clusters of negative perceptions (low Q-scores).
doi:10.1371/journal.pone.0068400.g004

has focused on the creation of mathematical models, such as those involved in the new economic geography of Krugman, Fujita and Venables [16–17], or on the establishment of empirical patterns, such as the knowledge spillovers documented by Glaeser and others [18–19].

Natural scientists, on the other hand, have a different focus than economists, but also rely on quantitative methods that do not incorporate the aesthetic features of the cities they study. Notable examples here include the study of the fractal growth of cities [20–21] and the study of allometric relations connecting population to a number of social and infrastructural variables [22]. Natural scientists have also been keen to develop automated data collection methods that use big data to study the statistical properties of citizens, such as their human mobility patterns [23–25] and social networks [26–30].

Table 1. Means and Standard Deviations of the Q-scores obtained for each city and question.

		Linz	Salzburg	Boston	NYC	Manhattan	Queens	Brooklyn
**Mean**	**Safety**	4.85	4.76	4.94	4.47	5.13	4.46	4.23
	**Unique**	4.84	5.04	4.77	4.46	5.21	4.26	4.31
	**Class**	5.01	4.89	4.97	4.31	5.17	4.22	4.06
**Standard Deviation**	**Safety**	0.80	0.88	1.48	1.41	1.25	1.35	1.44
	**Unique**	0.93	0.90	1.22	1.18	1.17	1.06	1.16
	**Class**	0.90	0.99	1.62	1.53	1.38	1.39	1.57

doi:10.1371/journal.pone.0068400.t001

Finally, the most direct connection between these two streams of literature is the work of Jane Jacobs [31–33] and the Broken Windows Theory (BWT) of Wilson and Kelling [34]. In "The Death and Life of Great American Cities" [31], Jacobs emphasizes the connections she observed between the physical environment of neighborhoods, and the social interactions between the citizens that inhabited them. "Death and Life" is well cited among architects and urban planners. Social scientists and economists, on the other hand, often build on Jacobs' later works, including "The Economy of Cities" [32] and "Cities and The Wealth of Nations" [33]. Hence, the literature bridge represented by Jacobs' work is largely due to her participation in both streams of literatures–and unfortunately – does not indicate a clear dialogue between them.

The Broken Windows Theory (BWT) of Wilson and Kelling [34], on the other hand, represents a more direct connection between the study of urban forms and social outcomes. In brief, the Broken Windows Theory suggests that evidence of environmental disorder, such as broken windows, litter and graffiti, can induce other kinds of disorder, like crime, and hence, policies that focus on the amelioration of minor offences can help fight more severe forms of criminal activity.

The BWT has also been politically influential. For instance, it was cited as a justification for New York City's quality-of-life initiative [35–36], an order-maintenance strategy that strictly enforces minor offenses, such as public drinking and turnstile jumping, as a way to prevent more substantial forms of crime, such as robbery.

Providing evidence to prove or disprove the BWT, however, has not been easy. In fact, several observational and longitudinal studies have argued in favor and against of the BWT [35–38]. Arguments against the BWT point to, among other things, the existence of spurious correlations in which underlying environmental features, such as liquor stores, can lead to both crime and disorder [36]. Arguments in favor of the BWT include experiments, like the ones performed by Keizer et al. [39]. Here the authors showed that in controlled settings, evidence of disorderly behavior, such as graffiti or supermarket carts left unattended in parking garages, were associated with an increase in the probability of people breaking other social norms, such as littering or stealing.

In recent years, the BWT has also been linked to health. For example, cases of gonorrhea in New Orleans have been shown to correlate more strongly with an index of neighborhood disorder than with an index of neighborhood poverty [40], and residents of disadvantaged neighborhoods in Illinois, where noise, graffiti and vandalism are more common, have been found to have worse health outcomes than residents of advantaged neighborhoods, even after controlling for individual level disadvantages [41].

All of these studies explore the link between people's perception of urban environments and social outcomes. Yet, the focus of this literature has been mainly on the association between crime and disorder, when this is only one of the many potential associations between the urban environment and social outcomes that can be of interest. In effect, urban landscapes are complex enough to demand a number of evaluative dimensions to be characterized [2], since beyond disorder places can look lively, modern, inspiring, classy, abandoned, congested, colorful or beautiful, among other things. These additional dimensions can be used to explore connections between aspects of urban perception and other social dimensions, such as entrepreneurship, civic engagement and high-school completion, among other things. To explore these connections, however, we need to extend our quantitative methods of urban perception beyond measures of disorder. In this paper, we show that it is possible to capture detailed information about other evaluative dimensions and show that this information can be used to characterize the inequality of cities with respect to these dimensions. Finally, inspired by the BWT, we validate the measures collected by comparing them with data on homicides for NYC.

Data and Methods

Data

We collected data on urban perception by using 4,136 geo-tagged images from four cities (# of images): New York City (1,706) and Boston (1,236) in the United States; and Salzburg (544) and Linz (650) in Austria, (Fig. 1A–D). Images from New York City (NYC) and Boston were sourced digitally from Google Street View while images from Linz and Salzburg were collected manually onsite. The images and dataset used in the study can be downloaded from (http://pulse.media.mit.edu/static/dataset/).

Perception data was collected using a website created for the study (Fig. 2A). Here users were shown two images, selected randomly from the dataset, and asked to click on one in response to one of three questions: "Which place looks safer?", "Which place looks more upper-class?", or "Which place looks more unique?". Users additionally had the option of indicating that both images were perceived as equal. The spatial location of images was not revealed to participants during the study.

We selected the phrasing "Which place looks more X?" because it reflected more accurately what could be evaluated from an image. We note that similar questions have been asked in preceding evaluative studies (*17*). 7,872 unique participants from 91 countries contributed a total of 208,738 votes and self-reported age and gender (SM and table 1s in File S2).

Some limitations of the data include the constrained amount of information that is captured in an image, since other sensory

Table 2. Comparison between the means and standard deviations of the urban perception recorded for each city and question.

Difference in Means

T-test for equal means with unequal variances.

Safety (p-values)

	Salzburg	Boston	New York	Manhattan	Queens	Brooklyn
**Linz**	0.0482**	0.1152	0.0000***	0.0004***	0.0000***	0.0000***
**Salzburg**		0.0015***	0.0000***	0.0000***	0.0000***	0.0000***
**Boston**			0.0000***	0.0201**	0.0000***	0.0000***
**New York**				0.0000***	0.9193	0.0001***
**Manhattan**					0.0000***	0.0000***
**Queens**						0.0028***

Unique (p-values)

	Salzburg	Boston	New York	Manhattan	Queens	Brooklyn
**Linz**	0.0001***	0.1547	0.0000***	0.0000***	0.0000***	0.0000***
**Salzburg**		0.0000***	0.0000***	0.0342**	0.0000***	0.0000***
**Boston**			0.0000***	0.0000***	0.0000***	0.0000***
**New York**				0.0000***	0.0003***	0.0033***
**Manhattan**					0.0000***	0.0000***
**Queens**						0.4156

Class (p-values)

	Salzburg	Boston	New York	Manhattan	Queens	Brooklyn
**Linz**	0.0317**	0.4844	0.0000***	0.0670*	0.0000***	0.0000***
**Salzburg**		0.2129	0.0000***	0.0019***	0.0000***	0.0000***
**Boston**			0.0000***	0.0291**	0.0000***	0.0000***
**New York**				0.0000***	0.2114	0.0002***
**Manhattan**					0.0000***	0.0000***
**Queens**						0.0535*

Difference in Variances

F-test

Safety (p-values)

	Salzburg	Boston	New York	Manhattan	Queens	Brooklyn
**Linz**	0.0257**	0.0000***	0.0000***	0.0000***	0.0000***	0.0000***
**Salzburg**		0.0000***	0.0000***	0.0000***	0.0000***	0.0000***
**Boston**			0.0633**	0.0003***	0.0216**	0.4562
**New York**				0.0091***	0.2913	0.4144
**Manhattan**					0.1296	0.0034***
**Queens**						0.1210

Unique (p-values)

	Salzburg	Boston	New York	Manhattan	Queens	Brooklyn
**Linz**	0.3764	0.0000***	0.0000***	0.0000***	0.0018***	0.0000***
**Salzburg**		0.0000***	0.0000***	0.0000***	0.0001***	0.0000***
**Boston**			0.2511	0.4196	0.0003***	0.1611
**New York**				0.8950	0.0037***	0.6196
**Manhattan**					0.0445**	0.8383
**Queens**						0.0252**

Class (p-values)

	Salzburg	Boston	New York	Manhattan	Queens	Brooklyn
**Linz**	0.0279**	0.0000***	0.0000***	0.0000***	0.0000***	0.0000***
**Salzburg**		0.0000***	0.0000***	0.0000***	0.0000***	0.0000***
**Boston**			0.0164**	0.0004***	0.0000***	0.3293
**New York**				0.0257**	0.0113**	0.2980
**Manhattan**					0.9122	0.0066***

Table 2. Cont.

**Difference in Means**	
**T-test for equal means with unequal variances.**	
**Queens**××	0.0024***

Significance thresholds * p<0.1 **p<0.05 ***p<0.01.
doi:10.1371/journal.pone.0068400.t002

channels that can affect perception, such as sound and smell, are absent in pictographic depictions. Also, variation in image quality (i.e. contrast, hue, saturation, brightness, tint and clarity), as well as the time of day, and weather conditions, can introduce additional sources of variation in the perceptions associated with a digital image. We therefore interpret the urban perception data collected through this method as a proxy for the perceptions elicited by the actual locations [2].

Finally, we note that the mapping between images and locations is not one-to-one. In fact, for a large number of locations we captured more than one image, by pointing the camera in two or more directions. Hence, many locations are characterized by more than one quantitative value –usually two. We captured more than one image for many locations to take into account the variability of using images that are not 360-degree representations of a place, but a 90-degree wedge.

Measures

We scored each image using the fraction of times it got selected over another image, corrected by the "win" and "loss" ratios of all images with which it was compared. This correction allowed us to adjust for the "strength of schedule" [42], since by chance some images were compared with others that were more likely to be selected favorably in pairwise comparisons. We define the win (W) and loss (L) ratios of image i with respect to question u as:

$$W_{i,u}=\frac{w_{i,u}}{w_{i,u}+l_{i,u}+t_{i,u}} \quad , \quad L_{i,u}=\frac{l_{i,u}}{w_{i,u}+l_{i,u}+t_{i,u}} \qquad (1)$$

where w is the number of times an image was selected over its paired image, l is the number of times that an image was not chosen over its paired image, and t is the number of times when an image was chosen as equal to its paired image. Using this, we define the Q-score for each image i and question u as:

$$Q_{i,u}=\frac{10}{3}\left(W_{i,u}+\frac{1}{n_i^w}\sum_{j_1=1}^{n_i^w} W_{j_1 u}-\frac{1}{n_i^l}\sum_{j_2=1}^{n_i^l} L_{j_2 u}+1\right) \qquad (2)$$

where niw is equal to the total number of images i was preferred over, nil is equal to the total number of images i was not preferred over, and where the first sum extends over j1, the images that image i was preferred over and the second sum extends over j2, the images that were preferred over i.

Equation (2) simply corrects an images win ratio ($W_{i,u}$) by adding the average win ratio of the images that it was selected over and by subtracting the loss ratio of the images that were selected over image i. By doing this, we incorporate information about the images that were paired together with each image. The numerical factors of 10/3 and 1 are used to scale the score to fit the range [0–10], and come from the theoretical minimum and maximums of the analytic expression *(2)* (see SM). In sum, a score of $Q=10$ represents the maximum possible score for safety, social-class or uniqueness, whereas $Q=0$ represents the minimum.

Robustness of Q

We test the inter-rater, or inter-observer reproducibility of Q, by comparing the scores obtained using the same number of images, but extracted from non-overlapping subsets of votes of size v. We do this using subsets containing up to 50% of the total votes, because it is not possible to construct non-overlapping subsets that are larger than 50% of the original sample. As our measure for inter-rater robustness (B), we use the average R2 of the Pearson correlation between rankings calculated using the same set of images, but a different set of votes. Formally, we define B as:

$$B(v)=\left(\frac{\sum_i (Q_i^1(v)-\langle Q^1(v)\rangle)(Q_i^2(v)-\langle Q^2(v)\rangle)}{\sigma_1\sigma_2}\right)^2 \qquad (3)$$

where $Q^1(v)$ and $Q^2(v)$ represent two sets of Q-scores calculated using disjoint sets of participants of size v, <> is used to indicate averages, and σ_1 and σ_2 are, respectively, the standard deviations of the Q-scores in the sets Q^1 and Q^2. We note that B is related to Cronbach's αand represents an estimate of the test-retest reliability of the method. A value of B = 100% indicates a perfectly robust ranking, since it would mean that the exact same set of Q-scores was obtained by using data collected from different people.

Figure 2B shows the average B obtained for subsets of different size v (thick line) for each question. We find that the behavior of B as a function of the sample size v is well approximated by:

$$B(v)=(1-exp(Bv^{\alpha}))^2 \qquad (4)$$

where α and β are fitting parameters ($R^2=99.7\%$ for safety, $R^2=99.9\%$ for social-class and $R^2=99.9\%$ for uniqueness). We use *(4)* to extrapolate the observed values (thin line Fig 2B) and infer the values expected for the totality of our dataset, finding that the 93,622 votes collected for the safety question (red square) results in $B=86.3\%$, the 70,157 votes available for the social-class question (blue square) results in $B=84.4\%$, and the 48,109 votes collected for uniqueness (green square) results in $B=56.0\%$.

Finally, we test the internal consistency of the perceptions collected by looking at their transitivity. We find that the overall level of transitivity of our data is high (86.76% for safety, 87.00% for social-class, and 83.34% for uniqueness).

As a rule of thumb, we find that between 22 and 32 votes per image are needed to produce a ranking with B>75% for each of the three questions.

One important concern that needs to be addressed here is the possible biases in the measures that might come from the demographic of participants that joined the online experiment. To test for this, participants were asked to self-report age and gender after contributing five clicks. Self-reporting was high, with

97.1% of the participants providing answers for age and gender. From these, 76.0% identified themselves as male and 21.1% as female. The median self-reported age was 28 years. Finally, participants were geo-located using their IP addresses and the 7,872 unique IP addresses were located in 91 countries.

We test the significance of possible biases by comparing the Q-scores estimated using different subsets of participants. We do this for participants' age (above and below the median), gender (male and female), and location (United States vs non-United States). As controls, we show the correlations obtained for random subsets of participants of the same size (Figures 1s, 2s and 3s in File S2). For example, we compare the correlation of the scores obtained for people older and younger than the median age of 28, with the correlation obtained for two disjoint random half-samples of participants. The same procedure was used to create controls for the correlations observed between groups of participant with different sex and for participants from US and non-US locations, as proxied by participants' IP-addresses. Overall, we find that the correlations obtained for groups of different demographics are not significantly lower than those obtained for the random controls, indicating that the results of our sample are not driven by biases in age, gender or location of the study's participants.

Results

We begin by asking whether perceptions of safety, class and uniqueness are perfectly collinear, or whether they have significant orthogonal components. Figures 3A–3C show typical images associated with high and low scores for safety, social-class and uniqueness. Places perceived as safe are also more likely to be perceived as upper-class (Fig. 3D $R^2 = 68.94\%$, p-value<0.0001) and unique (Fig. 3E $R^2 = 35.32\%$, p-value<0.0001), yet, their orthogonal components ($1-R^2$) are relatively large. This allows us to identify images matching particular combinations of evaluative criteria, such as images where the perception of safety matches that of social-class (Fig. 3D–I and 3D–III) and where social-class and safety are inversely related (Fig. 3D–II and 3D–IV). Figure 3F shows the analysis for the remaining combination of social-class and uniqueness ($R^2 = 37.04\%$, p-value<0.0001). Together, these results show that data collected through this method can be used to identify images satisfying combinations of criteria, and therefore can distinguish between the perceptions of safety, social-class and uniqueness.

Next, we use Q to measure the contrast or inequality of urban perception. We begin this by asking: how wide is the range of perceptions elicited by the images of one city vis-a-vis another? Figure 4A shows the distribution of scores characterizing each image, for each city and question (values are reported in Table 1). Here, we see that images in Boston and NYC are distributed over a wider range of values. Yet, since we have considerably more images for Boston and NYC, than for Linz and Salzburg, we compare the standard deviations of these distributions (σ), rather than their range. We do this because the standard deviation of a distribution is independent of sample size and provides a good comparator to measure the dispersion of the Q-scores calculated for each city. Moreover, the distribution of Q-scores for each question is close to normal (see SM and Figure 4s in File S2).

Table 2 compares the means and standard deviations of each city and question using, respectively, a t-test to compare the means of distributions with different variances, and an F-test. The F-Test allows us to assess whether the difference between the standard deviations of two distributions is significant, after taking into consideration their sample size [43]. We find that the standard deviations of the distribution for Boston and NYC are considerably larger than those for Linz and Salzburg, even when there are no significant differences in the mean (for example with the means of Linz and Boston for social-class). This suggests that Boston and NYC are perceptually more unequal, since the average gap of the evaluative response between images is larger in NYC and Boston than in Linz and Salzburg. Moreover, we note that the standard deviation measured for NYC is not statistically larger than the one measured for Queens and Brooklyn, when it comes to the perception of safety and class.

Next, we study the segregation of urban environments by asking if the places associated with similar perceptions of safety, social-class and uniqueness co-locate, and if so, to what extent. In principle, a wider range of values is observed for Boston and NYC, but these could be spatially intermixed rather than clustered. To measure the spatial segregation of perceptions we use Moran's I statistic [44]. Values of I range from -1 to 1. A value of -1 indicates perfect anti-correlation (e.g. a checkerboard), whereas a value of 1 indicates that similar values are perfectly clustered. The null-hypothesis of I is complete spatial randomness and produces values near 0. Moran's I statistic, however, cannot be used directly to make statistical inferences, since its significance depends on the sample size. Hence, we normalize the Moran I scores for each city by subtracting the city's average and dividing it by its standard deviation (creating a z-score). We also control for differences in sample size by randomly down-sampling the data for Boston, NYC and Linz to match the 544 points available for Salzburg. This guarantees that all datasets have the same sample size and ensure that variations are not due to differences in the number of points considered.

Figure 4B shows the z-scores associated with Moran's I for each city and question (see Table 3s in File S2 for p-values). In general we find that all cities exhibit positive spatial autocorrelation, with Boston and New York having higher z-scores than Linz and Salzburg. These results suggest that the American cities studied have more segregated neighborhoods than the Austrian cities of Linz and Salzburg. To explore this further, we measure the length of the spatial autocorrelation using the autocorrelation function:

$$A(|\vec{d}|) = \frac{\langle (Q(\vec{r}) - <Q>)(Q(\vec{r}+\vec{d}) - <Q>) \rangle}{\sigma_2} \qquad (5)$$

Figure 4C shows the autocorrelation function *(5)* for each city and for the three NYC boroughs of Manhattan, Queens and Brooklyn. We note that since many locations contain more than one image –images captured with the camera pointing in a different direction–$A(0)<1$, since this represents the correlation between images captured in the same location but with a different heading. Finally, we measure the correlation length of each of these using:

$$A = \mu e^{-\nu|\vec{d}|} + \eta \qquad (6)$$

where μ, η and η are fitting parameters. η is included to capture the negative correlations observed for large values of $|\vec{d}|$ (>5 [km]). To ease interpretation, we define l as the distance $|\vec{d}|$ at which $A(|\vec{d}|) = 0$. To avoid measurement errors due to binning, we take the average l calculated empirically using a series of bins ranging from 100 [m] to 1000 [m], for every 100 [m].

NYC is found to be the city with the largest autocorrelation length, having all $l>4.75$ [km]. Boston's mean autocorrelation length for the three questions is $l>2.00$ [km] whereas Linz and

Table 3. Getis Spatially Filtered Regression including variables for demographic and urban perception.

	Getis Spatially Filtered Regression. Dependent Variable -> Log (Number of Homicides in Zip Code +1)															
	**DEMOGRAPHICS**								**URBAN PERCEPTION**							
	**Population and Area**				**Income and Age**				**Safety**	**Class**						
MODEL 1	**Log(Pop)***	**L_Log (Pop)**	**Log (Area)***	**L_Log (Area)**	**Log (Income)***	**L_Log (Income)**	**Log10 (Age)***	**L_Log10 (Age)**	**Qsafety***	**L_Qsafety**	**SQ safety***	**L_SQ safety**	**Qclass***	**L_Qclass**	**SQ Class***	**L_SQClass**
**Coefficient**	0.262**	0.188*	0.569***	−0.419	−0.954***	−0.453	−1.559**	−14.89**								
**t-statistic**	2.298	2.798	5.416	−0.510	−4.820	−0.345	−2.486	−2.216								
**p-value**	0.024	0.075	0.000	0.611	0.000	0.731	0.015	0.029								
MODEL 2															**R2**	69.9%
**Coefficient**	0.868***	−0.599	−0.181	−0.220					−0.181***	−1.033***	−0.109	−0.416				
**t-statistic**	6.362	−0.453	−1.132	−1.465					−3.655	−2.746	−1.269	−0.745				
**p-value**	0.000	0.651	0.260	0.146					0.000	0.007	0.208	0.458				
MODEL 3															**R2**	47.8%
**Coefficient**	0.833***	−1.046	−0.208	−0.260**									−0.180***	−0.713**	0.045	0.728
**t-statistic**	6.115	−0.711	−1.316	−1.742									−3.801	−2.262	0.053	1.292
**p-value**	0.000	0.479	0.191	0.085									0.000	0.026	0.600	0.200
MODEL 4															**R2**	48.3%
**Coefficient**	0.837***	−1.737	−0.222	−0.257*					−0.073	−0.856	−0.213**	−2.480**	−0.144	0.118	0.189*	2.560***
**t-statistic**	5.995	−1.171	−1.407	−1.733					−0.681	−0.073	−1.976	−2.535	−1.379	0.116	1.732	2.696
**p-value**	0.000	0.245	0.163	0.086					0.497	0.484	0.051	0.013	0.171	0.908	0.086	0.008
MODEL 5															**R2**	52.9%
**Coefficient**	0.392***	0.347***	0.481***	0.936	−1.183***	−2.252	−1.545***	−22.45***	−0.035	−2.717***	−0.210***	−1.103	0.033	3.511***	0.180**	1.259*
**t-statistic**	3.172	2.957	4.774	0.803	−5.642	−1.228	−2.686	−3.033	−0.465	−3.089	−2.732	−1.341	0.444	4.487	2.336	1.917
**p-value**	0.002	0.004	0.000	0.424	0.000	0.223	0.009	0.003	0.643	0.003	0.008	0.183	0.658	0.000	0.020	0.058

The dependent variable is the logarithm–in base 10–of the number of homicides in a zip code plus one. The plus one was added to include zip codes in which the number of homicides is zero. Significance thresholds are: * $p<0.1$ ** $p<0.05$ *** $p<0.01$.
doi:10.1371/journal.pone.0068400.t003

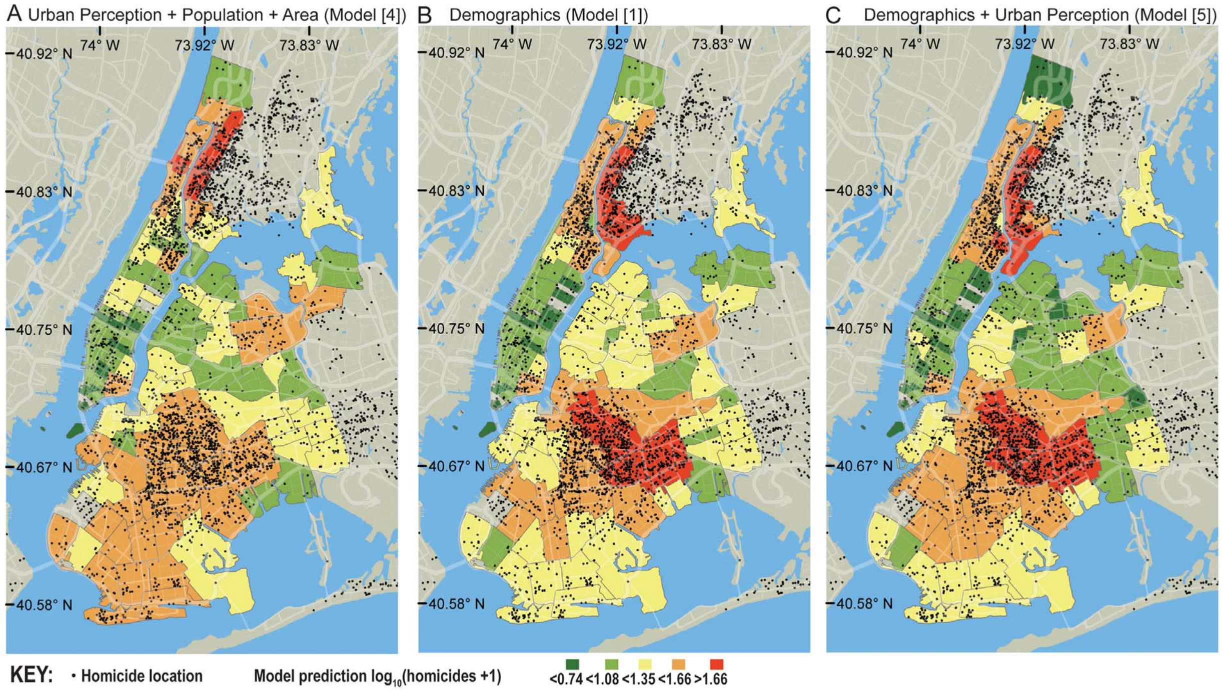

Figure 5. Urban perception and violent crime. A Comparison between the location of crimes in NYC and the predictions of urban perception, area and population (model [4]). **B**. Demographics (model [1]). **C**. All variables (model [5]).
doi:10.1371/journal.pone.0068400.g005

Salzburg have characteristic lengths of 1.6 [km] or less. This shows that locations associated with similar perceptions form larger spatial clusters in NYC (Figures 4 D–F) and Boston than in Linz and Salzburg. Finally, we note that the NYC boroughs of Manhattan, Brooklyn and Queens all exhibit strong autocorrelation, with lengths only slightly smaller than that of NYC. This suggests that the measures obtained for NYC also hold for smaller spatial scales in that city, yet a detailed evaluation of the association between the segregation of urban perception and city size will require data on a larger number of cities.

Urban perception and violent crime

Finally, we use homicide data for NYC to look at the correlation between the urban perception of inequality and homicides. We note from the start that our intention is not to make a causal statement, but simply to use this correlation to validate the value of the information contained in our measures of urban perception. Because of the spatial nature of the dataset, we use Getis Spatially Filtered Regression (GSFR) [45–46], rather than an Ordinary Least Square (OLS) regression. In spatial datasets is not appropriate to use OLS regressions because of the existence of spatial auto correlations. In other words, the fact that neighboring cells are characterized by similar values violates the independence assumption needed to perform an OLS. So, an OLS is only justified if the residuals of the OLS regression are NOT spatially auto-correlated. This is because the autocorrelation of the residuals would indicate the existence of unexplained spatial variation, and therefore, the existence of a missing variable. In statistics, we would say that in this case the model is under-specified.

GSFRs solve this problem by using a transformation that filters out the spatial component of each variable x, into two estimates: one capturing the spatial variation of the variable (L_x), and the other capturing the local variation of this variable remaining after the spatial variation has been removed (x^*). For each location i, and variable x, these variables are defined as:

$$x_i^* = \frac{x_i S_i}{G_i(n-1)} \quad (7)$$

$$L_{x_i} = x_i - x_i^* \quad (8)$$

where $S_i = \Sigma_j s_{ij}$ is the sum of the spatial weights used to characterize the spatial proximity between data points (in our case 1/distance between locations i and j), n is the number of locations considered and

$$G_i = \frac{\sum_j w_{ij} x_j}{\sum_j x_j} \text{ for j}\neq\text{I} \quad (9)$$

Finally, a GSFR regression is an OLS regression where each variable x is replaced by its spatially filtered x^* and varying component L_x. More details about this statistical technique can be found in [45]. To illustrate what the method doe consider the income of a zip code. This is a variable that is certainly spatially autocorrelated, since rich zipcodes are more likely to locate next to other rich zipcodes. Instead of incorporating income as a variable, a GSFR will incorporate an *income** variable, which would be the income of a zip code that is not explained by the incomes of nearby zip codes, and a L_{income} variable, that would capture the spatial variation of income across zip codes.

Table 3 shows the results of a GSFR where the dependent variable is the logarithm of the number of homicides in a NYC zip code recorded between 2003 and 2011. We note that the Google

Street View API does not provide information for the date and time the images were captured. As explanatory factors we use the average incomes of households in the zip-code, population, area, age and four urban perception variables: the average Q-score for safety and class (Q_{safety}, Q_{class}), and their respective standard deviations (SQ_{safety}, SQ_{class}) calculated for each zip-code. Formally, the regression takes the form:

$$log_{10}(Homicides+1)=B_1\vec{x}^{*}+B_2\vec{L}_x+\varepsilon \quad (10)$$

Table 3 presents 5 different specification of the statistical model. All models include the population and area of a zip code, since these are obvious correlates of crime. Model 1 includes also income and age. Model 2 adds the perception of safety, while model 3 includes the perception of class. Model 4 includes the perception of class and safety, but no information on age or income. Finally, model 5 includes all variables –population, area, income, age, average perception of safety, average perception of class, standard deviation in the perception of safety, and standard deviation in the perception of class. We note that for the full specification of our model (model [5]), we find no spatial correlations among the residuals (Moran's I z-score $=-0.23$, p-value $=0.82$), indicating that the model is not underspecified and can be used for statistical inference. Hence, the results cannot be interpreted as the result of a missing variable, such as policing or race [45–46].

Model 5 explains nearly 80% of the variation of homicides across zip codes. This correlation is 10% larger than what is explained by income, age, population and area alone –from 69.88% (model [1]) to 79.36% (model [5])). The increase is statistically significant ($F=5.3$, p-value$<1.8\times10-^{5}$), and indicates that the measures of urban perception contain information on the location of homicides that is not contained in income.

Overall, we find that in the full model (model [5]), the spatial components (LQ_{safety}, LQ_{class}), and not the local intensity components (Q_{safety}*, Q_{class}*) are statistically significant meaning that the spatial variation of urban perception across the city, is what correlates significantly with the location of homicides. Moreover, we find that the local spread of perceptions within a zip-code (SQ_{class}*, SQ_{safety}*) correlates with the number of homicides. These results are consistent in the sense that spatial variations for the perceptions of safety and class (rather than their absolute values) correlate with violent crime, after introducing the control variables. A visual comparison of the statistical models presented in table 3 is presented in figure 5.

Finally, we notice that the regression coefficients of the safety variables are negative (safer looking, less crime), whereas those of class are positive (classier looking, more crime). As expected, coefficients of safety and class are negative when introduced individually (models [2] and [3]), but the one for class reverse signs when we control for safety (models [4] and [5]). We interpret the opposite signs of these coefficients as evidence that the orthogonal component between class and safety (Figure 3D) carries important information, since it indicates that violent crime occurred in places that look relatively more upper class after controlling for their perception of safety.

Conclusions

The way a city looks is of central importance for the daily experience of billions of city-dwellers. Yet until now, the availability of data about urban perception has been limited, and so has our ability to compare cities with respect to them. In this paper, we presented a method to measure urban perception and found that the cities of Boston and NYC differ from the Austrian cities of Linz and Salzburg in two important dimensions. First, the perceptions recorded for the cities of Boston and NYC are distributed more broadly than the perceptions elicited by the images from the two Austrian cities of Linz and Salzburg. Second, positive and negative perceptions cluster more strongly in the two American cities, than in their European counterparts. This means that the recorded gap between "good" and "bad" neighborhoods is larger in NYC and Boston and that both positively evaluated and negatively evaluated images cluster more in these American cities than in their Austrian counterparts. Finally, we showed that the inequality of perceptions helps explain the location of violent crime in a NYC zip code, even after controlling for income, population, area and age.

As the world gears towards building cities for hundreds of millions of individuals, the imperative of understanding cities becomes ever more important [3]. Therefore, there is a strong need to create quantitative bridges that can help us link urban perception with other social, political, economic and cultural aspects of cities. In this paper, we present a method that can be used to quantify urban perception and have applied it to the study of a few cities and questions. Although the method offers an important improvement in throughput over previous studies, its ability to collect data is limited to web traffic and participation. Because of this, future iterations will need to consider the use of a combination of crowdsourcing and machine learning tools to extend the patterns captured by the online participation data to higher resolution and different latitudes. Moreover, future studies might also explore the perceptual biases associated with the measurement technique presented in this paper, as well as support the development of techniques that can help identify the features that determine the evaluative responses recorded. Ultimately, the goal of this study – and those similar to it – is to contribute to our understanding of the urban environments that we have built, with the goal of improving them, and their ability to include their citizens, while also informing the construction of future cities.

Supporting Information

File S1 Q scores.
(XLS)

File S2 Supplementary material.
(DOCX)

Acknowledgments

We would like to thank Deepak Jagdish for compiling and organizing the dataset before release. We would also thank Kiran Bhattaram, David Gelvez, Sep Kamvar, Kent Larson, Evan Marshall, Shahar Ronen, Alex Simoes, Paul Sawaya, Michael Xu and Michael Wong for their comments and expertise. We acknowledge support from the MIT Media Lab consortia, and the ABC Career Development chair.

Author Contributions

Conceived and designed the experiments: CAH. Performed the experiments: PS. Analyzed the data: CAH PS. Contributed reagents/materials/analysis tools: CAH PS KS. Wrote the paper: CAH KS PS. Original Idea: CAH.

References

1. Lynch K (1960) The image of the city (Vol. 1). MIT press.
2. Nasar JL (1997) The Evaluative Image of the City. Sage Publications.
3. Rybczynski W (2010) Makeshift metropolis: ideas about cities. Scribner.
4. Robinson CM (1909) Modern civic art: or, The city made beautiful. GP Putnam's sons.
5. Howard E, Osborn FJ (1965) Garden cities of to-morrow (Vol. 23). The MIT Press.
6. Scott JC (1998) Seeing like a state: How certain schemes to improve the human condition have failed. Yale University Press.
7. Devlin K, Nasar JL (1989) The Beauty and the Beast, Journal of Environmental Psychology 9:333–344.
8. Peterson GL (1967) A Model of Preference, J Regional Sci 7:19–31.
9. Schroeder HW, Anderson LM (1984) Perception of Personal Safety in Urban Recreation Sites, Journal of Leisure Research 16:178–194.
10. Herzog TR, Kaplan S, Kaplan R (1976) The Prediction of Preference for Familiar Urban Places, Environment and Behavior 8:627–645.
11. Roth M (2005) Online Visual Landscape Assessment Using Internet Survey Techniques Trends in online landscape architecture. Proceedings at Anhalt University of Applied Sciences, 121–130.
12. Wherrett JR (2010) Creating Landscape Preference Models Using Internet Survey Techniques, Landscape Research 25:79–96.
13. Milgram S (1976) Psychological maps of Paris. Environmental psychology: People and their physical settings, 104–124.
14. Wilson RL (1962) Livability of the city: attitudes and urban development. Urban Growth Dynamics, 359–399.
15. Chapin FS, Weiss SF (1966) Urban Growth Dynamics in a Regional Cluster of Cities.
16. Krugman P (1998) What's new about the new economic geography?. Oxford review of economic policy, 14(2):7–17.
17. Fujita M, Krugman P (2003) The new economic geography: Past, present and the future. Papers in regional science 83(1):139–164.
18. Glaeser EL, Kallal HD, Scheinkman JA, Shleifer A (1992) Growth in Cities. Journal of Political Economy, 100(6).
19. Ellison G, Glaeser EL (1997) Geographic Concentration in US Manufacturing Industries: A Dartboard Approach. Journal of Political Economy, 105(5):889–927.
20. Batty M (2007) *Cities and complexity: understanding cities with cellular automata, agent-based models, and fractals*. The MIT press.
21. Batty M, Longley PA (1994) *Fractal cities: a geometry of form and function*. Academic Press.
22. Bettencourt LMA, Lobo J, Helbing D, Kuhnert C, West GB (2007) Growth, innovation, scaling, and the pace of life in cities. Proceedings of the National Academy of Sciences (PNAS) 104(17):7301–7306.
23. González MC, Hidalgo CA, Barabási AL (2008) Understanding Individual Human Mobility Patterns. Nature 435:779–782.
24. Brockmann D, Hufnagel L, Geisel T (2006) The scaling laws of human travel, Nature 439(7075):462–465.
25. de Montjoye YA, Hidalgo CA, Verleysen M, Blondel VD (2013) Unique in the Crowd: The privacy bounds of human mobility. Scientific reports 3.
26. Eagle N, Pentland A, Lazer D (2009) Inferring Social Network Structure using Mobile Phone Data, Proceedings of the National Academy of Sciences (PNAS) 106 (36):15274–15278.
27. Onnela JP, Saramaki J, Hyvonen J, Szabo G, Lazer D, et al. (2007) Structure and tie strengths in mobile communication networks. Proceedings of the National Academy of Science 18:7332–7336
28. Hidalgo CA, Rodriguez-Sickert C (2008) The dynamics of a mobile phone network. Physica A: Statistical Mechanics and its Applications, 387(12):3017–3024.
29. Palla G, Barabasi AL, Vicsek T (2007) Quantifying social group evolution, Nature 446 (7136): 664–667.
30. Eagle N, Macy M, Claxton R (2010) Network Diversity and Economic Development, Science 328(5981):1029–1031.
31. Jacobs J (1961) The death and life of great American cities. Vintage.
32. Jacobs J (1970) The economy of cities. The economy of cities.
33. Jacobs J (1985) Cities and the wealth of nations: Principles of economic life. New York: Vintage Books.
34. Kelling GL, Wilson JQ (1982) Broken windows. Atlantic monthly, 249(3):29–38.
35. Bratton W, Kelling G (2006) There are no cracks in the broken windows. National Review, 28.
36. Harcourt BE (1998) Reflecting on the Subject: a Critique of the Social Influence Conception of Deterrence, the Broken Windows Theory, and Order-Maintenance Policing New York Style, Michigan Law Review 97:291–389.
37. Harcourt BE, Ludwig J (2006) Broken windows: New evidence from New York City and a five-city social experiment. The University of Chicago Law Review: 271–320.
38. Jean PKS (2007) *Pockets of crime: Broken windows, collective efficacy, and the criminal point of view*. University of Chicago Press.
39. Keizer K, Lindenberg S, Steg L (2008) The spreading of disorder. Science, 322(5908):1681–1685
40. Cohen D, Spear S, Scribner R, Kissinger P, Mason K, Wildgen J (2000) "Broken windows" and the risk of gonorrhea. American Journal of Public Health, 90(2):230.
41. Ross CE, Mirowsky J (2001) Neighborhood disadvantage, disorder, and health. Journal of health and social behavior, 258–276.
42. Park J, Newman ME (2005) A network-based ranking system for US college football. Journal of Statistical Mechanics: Theory and Experiment, 2005(10):10014.
43. Lomax RG (2007) An introduction to statistical concepts.
44. Moran PA (1950) Notes on continuous stochastic phenomena. Biometrika, 37(1/2):17–23.
45. Getis A (1990) Screening for spatial dependence in regression analysis. In Papers of the Regional Science Association 69(1):69–81 Springer-Verlag.
46. Anselin L, Getis A (2010) Spatial statistical analysis and geographic information systems. In Perspectives on Spatial Data Analysis 35–47. Springer Berlin Heidelberg.
